# A randomized controlled study of an e-learning program (YURAIKU-PRO) for public health nurses to support parents with severe and persistent mental illness and their family members

**DOI:** 10.1186/s12912-022-01129-0

**Published:** 2022-12-05

**Authors:** Masako Kageyama, Keiko Koide, Ryotaro Saita, Riho Iwasaki-Motegi, Kayo Ichihashi, Kiyotaka Nemoto, Setsuko Sakae, Keiko Yokoyama

**Affiliations:** 1 Osaka University Institute of Advanced Co-Creation Studies, 1-7 Yamadaoka, Suita, Osaka 565-0871 Japan; 2 Department of Health Promotion Science, Osaka University Graduate School of Medicine, 1-7 Yamadaoka, Suita, Osaka 565-0871 Japan; 3 Department of Medical Innovation, Osaka University Hospital, 2-2 Yamadaoka, Suita, Osaka 565-0871 Japan; 4 Section of Public Health Nursing Research Department of Health Promotion, National Institute of Public Health, 2-3-6 Minami Wako, Saitama, 351-0197 Japan; 5 Department of Neuropsychiatry, University of Tokyo Hospital, 7-3-1 Hongo, Bunkyo, Tokyo 113-8655 Japan; 6 Department of Psychiatry, Faculty of Medicine, University of Tsukuba, 1-1-1 Tennodai Tsukuba, Ibaraki, 305-8575 Japan; 7 Department of Social Design, Faculty of Sociology, St. Andrew’s University, 1-1 Manabino, Izumi, Osaka 594-1198 Japan; 8 Department of Nursing, Faculty of Nursing, Yokohama Soei University, 1Miho-cho, Yokohama City, Kanagawa, 226-0015 Japan

**Keywords:** Parental mental illness, Professional development, Public health nurses, Family-focused intervention, Child-raising, e-learning program

## Abstract

**Background:**

Supporting parents with severe and persistent mental illness (SPMI) requires knowledge, skills, and a positive attitude toward parenthood. We developed a Japanese e-learning program for public health nurses (PHNs) to enable them to support parents with SPMI and their family members. This study aimed to evaluate the effectiveness of the program in improving the knowledge, skills, attitudes, and self-efficacy of PHNs in supporting them.

**Methods:**

A three-hour video-based e-learning program was developed. A randomized controlled trial was conducted with 176 PHNs responsible for maternal and child health in Japan. The outcome measures included the Sense of Coping Difficulty/Possibility Scale, skills to support people with SPMI, and achievement of program goals. Outcome data were collected at three time points during the study: baseline (T1), post-intervention (T2), and one month after T2 (T3) using self-administered electronic questionnaires. Outcome measures were assessed by comparing the two groups at the endpoint (T3) using t-tests and ANOVA. Effectiveness over time was assessed using a mixed model for repeated measures, with group and time interactions as fixed effects.

**Results:**

The study participants were randomly allocated to two groups:89 in the intervention group, and 87 in the control group. The total score and the scores in the two subscales of the Sense of Coping Difficulty/Possibility Scale in the intervention group at T3 were significantly higher than those in the control group, as shown by the t-test and ANOVA (all *p*<0.001). The Sense of Coping Difficulty subscale had a large effect size (Cohen’s d=1.27). The analysis of the results of a mixed model for repeated measures showed that the group and time interactions on all outcome measures were not significantly different at T1 but were significantly different at T2 and T3.

**Conclusions:**

The program was effective one month after its completion, particularly in reducing PHNs’ difficulties in supporting parents with SPMI.

**Trial registration:**

UMIN000045765, November 1, 2021.

## Background

According to an international survey by the World Health Organization, the lifetime prevalence of mental illness ranges from 12% to 47% (18% in Japan) [1]. Some individuals with mental illness become parents. The percentage of their becoming parents is comparable to the percentage of people without mental illness [2, 3], and one in seven children has a parent with mental illness [4, 5]. Some parents with mental illness raise their children without professional support. However, some people with severe and persistent mental illness (SPMI) who use adult psychiatric services become parents[6] and require professional support to raise their children. In a national survey in Japan, 15.5% of people with SPMI and a disability certificate under the age of 65 lived with a child or children [7]. The Japanese government has been working to deinstitutionalize patients with SPMI [8], and it is expected that more people will live in the community and become parents.

The symptoms and cognitive impairment of parental SPMI can create difficulties in maternal-child interactions, household chores, and childcare [9–13]. Parental SPMI is a risk factor for child maltreatment [14]. Programs supporting parents with SPMI have been developed, and several systematic and scoping review articles have been published on these programs [15–18]. A recent scoping review of parent support programs over the past 20 years [18] found 34 programs. It also identified the intervention elements often found in programs, such as improved parenting skills and long-term coordinated support for the entire family.

Nurses play a crucial role in supporting parents with SPMI [19]. In Japan, public health nurses (PHNs) in the maternal and child health field of local governments play an important role in providing direct support to parents and children from pregnancy to preschool age [20]. Despite parents suffering from SPMI, direct support is provided in collaboration with the child abuse and mental health fields when needed [20]. Although parental support programs for SPMI have mainly been developed in Europe, the U.S., and Australia [18], it is difficult for PHNs in Japan to introduce these programs into their usual care. Therefore, it is necessary to improve their skills and enable them to provide direct care through their usual care without introducing special programs. However, PHNs in Japan find it difficult to support parents with SPMI [21]. Nurses in Japan working in the community face challenges in building therapeutic relationships with parents with SPMI and collaborating with multiple agencies [21–24]. These skills need to be improved as failure to properly provide support can lead to difficulties, such as refusal to support or inability to provide timely support for them [21–24].

Additionally, a systematic review article dealing with parents’ experiences with SPMI regarding the care they received [25] revealed that coordinated and psychological support for parents was inadequate and perceived as not meeting their support needs and stigma. Nurses must provide sufficient support to meet parents’ needs in collaboration with multiple agencies to reduce stigma.

As in Japan, working with parents with SPMI and their families in other countries can be perceived as difficult, complex, and challenging for nurses [26]. Vives-Espelta et al.[26] published the first review article focusing on nurses’ views and practices regarding parental SPMI in 2022. According to this article, family-focused practices, interagency collaboration, and building therapeutic relationships are essential for nurses. To implement family-focused practice, the article suggested the need for nurses to develop knowledge about parents with SPMI and to increase self-efficacy in supporting parents in a community setting [26].

Family-focused practices emphasize family as a unit of attention [27]. In supporting parental SPMI, the importance of supporting the parent and entire family, including the child(ren) and partner, was noted [18, 19, 26, 28, 29]. Involvement with the entire family means that there are many agencies involved with each family member and coordinated support through inter-agency collaboration. Communication with other services is an important skill in inter-agency collaboration [30].

Building therapeutic relationships with parents and their family members requires professionals to have positive attitudes toward them [31]. However, more than 50% of the studies included in the review article on nurses’ views and practices regarding parental SPMI included the concept of stigma [26]. Stigmatizing attitudes were related to more negative views on parenthood and fewer interventions to support parental roles [32]. In the training framework for delivering professional development programs to support parental SPMI [33], reducing stigma is necessary at the beginning of the training program.

Therefore, it is important for nurses to improve the knowledge and skills necessary to support parents with SPMI [26] and reduce stigma and negative attitudes toward parents [26, 34, 35]. Family-focused practices [18, 19, 26, 28, 29], building therapeutic relationships [21, 23, 26], and interagency collaboration [18, 21, 26, 28] are important skills for supporting parental SPMI. Nurses should develop these skills and increase their self-efficacy to support parents with SPMI and their family members in the community.

E-learning is rapidly gaining popularity as a means of continuing education for nurses, and their satisfaction with such programs is high [36]. The literature has noted considerable advantages of e-learning for nurses’ skills, knowledge, attitude, and self-efficacy [36]. Disadvantages have also been reported related to interactions and technical education [36, 37]. As a result of the COVID-19 outbreak, e-learning resources have become increasingly important [38].

Digital storytelling has also been used in nursing education [39]. It comprises short videos that combine standalone and first-person narratives, with the inclusion of multimedia [39]. Storytelling in nursing improves nurses’ interactions with patients because it reveals preconceived notions and stereotypes about patients to improve their self-awareness [40]. Digital storytelling can therefore be expected to help nurses become aware of the stigma they face by conveying the patient's perspective and can change their negative attitudes to positive ones.

E-learning programs are available for professional development in the area of parental mental illness [33]. The two e-learning training programs, “Keeping Families and Children in Mind” [41] and “Let’s Talk about Children” [42] were evaluated with the pre-post evaluation. These e-learning training programs use digital storytelling and include family-focused practices and reported effects such as knowledge, skills, and confidence with regard to mental health professionals [41, 42]. However, these programs are designed for mental health professionals and not for nurses in charge of maternal and child health. Moreover, the information available for support and the role of support organizations differ from country to country; therefore, programs appropriate for Japan are required.

This study aimed to evaluate the effectiveness of a Japanese e-learning program for PHNs in supporting parents with SPMI and their family members.

## Methods

### Design

A randomized controlled trial (RCT) was conducted to test the effectiveness of the program. The study participants were randomly divided into intervention and control groups by stratified allocation based on their years of experience. The program was offered online, and only the intervention group could view it multiple times and at their own convenience during the six-week period. The outcome measures were evaluated by using self-administered electronic questionnaires from the Research Electronic Data Capture (REDCap) to compare the two groups at three time points during the study: pre (T1), post (6 weeks after T1; T2), and one month after T2 (T3). Ethical considerations allowed the control group to participate in the same program after the final T3 response. The trial findings were reported in a subsequent publication, according to the Consolidated Standards of Reporting Trials (CONSORT).

### Study participants

The inclusion criteria for the study were full-time PHNs in charge of maternal and child health who worked at city health centers in Japan, and had less than ten years of experience as PHNs. The exclusion criteria consisted of those on whom electronic questionnaires or video-viewing operations could not be conducted. Information about the study was mailed to all 833 cities nationwide and the applicants applied individually.

The sample size was determined with reference to the effect size of a similar e-learning program for practitioners [41]. A two-sample t-test with an effect size of 0.45, α=0.05, and β=0.20, yielded the required study population of 158 subjects. The sample size was set to 176, considering a dropout rate of approximately 10%. This sample size represented 4.4% of the approximately 4,000 PHNs eligible to participate in the study.

### Outcome measures

#### Sense of coping difficulty/possibility scale

This scale refers to analogous concepts of self-efficacy, that is, the efficacy expectation of the feeling that professionals can deal with families in difficult situations [43, 44]. In a previous study, higher self-efficacy of PHNs was related to better support skills regarding maternal-infant mental health [45]. The scale has been tested for reliability and validity and consists of two subscales: Sense of Coping Difficulty and Sense of Coping Possibility [43, 44]. In this study, the total score as a primary outcome measure and the scores of the two subscales as secondary outcome measures were used for the evaluation. The Sense of Coping Difficulty subscale consists of five items, these are: “It is difficult to intervene because I feel that intervening will isolate children or families from the community,” “I do not know how to get involved if the children or families refuse to meet with me,” “I do not know how to get involved because the children or families have special problems,” “It is difficult to change children or families,” and “I do not know how to respond if I want to support but the children or families will not respond.” The Sense of Coping Possibility subscale consists of five items, these are: “I think I can bring out the best in children and families by changing the way I interact with them,” “I can communicate with uncooperative children and families as needed,” “I think there are many aspects of children and family issues that can be discussed and devised by those around us,” “I can notice that a child or family member has ‘changed’ even if it is a small thing,” and “I can notice the good things about a child or family member even if it is a small thing.” The researchers made use of the two subscales to reflect on PHNs’ skills in relation to providing direct support to the whole family and building therapeutic relationships. The researchers further believe that the Sense of Coping Possibility subscale could determine changes in PHNs' attitudes because it reflects positive attitudes toward children and families.

In this study, the following items were asked about supporting parents in raising their children. For each item, the total score was given in the format of “not at all disagree” (1), “disagree” (2), “agree” (3), or “agree very much” (4). All items on the Sense of Coping Difficulty subscale were replaced with reversed items. Higher scores indicate that they felt less challenged and more capable of coping with supporting parents in raising their children. In summary, higher scores indicate higher self-efficacy.

#### Skills related to mental health issues

The validity of the unidimensional variable of the four items to self-evaluate the skills in dealing with mental health issues was confirmed [46]. In this study, the object score was reduced to a one-dimensional scale by using principal component analysis (PCA). Of the original four items, three related to knowledge, support skills, and support experience were used, except for one item related to the experience of attending training on mental health issues because it was related to program participation in this study. The researchers believe that these items can be used to measure overall mental health, knowledge, and skills of PHNs.

#### Achievement on program goals

The achievement level of the six program goals was assessed on a seven-point scale from “not at all” (1 point) to “very much” (7 points) in terms of how well PHNs could explain their knowledge and skills.

#### Basic characteristics

In addition to age and gender, this study identified support experiences, which can be related to the practice of support [28]. Considering previous studies on PHNs [45, 46], the researchers obtained years of experience as PHNs in charge of mental health services, maternal and child health, supporting parents with SPMI, learning about support for parents with SPMI, and supporting people with SPMI who do not raise their children.

#### Process and feasibility evaluation measures

The number of times each module was viewed is reported. To evaluate feasibility based on the length of the program, the participants in the intervention group were asked to choose among long, just right, or short.

### Randomization and blinding

Following the participants’ applications, entries were made in the order in which they were confirmed to have been sent and received through e-mail. The participants were automatically divided into two groups using REDCap, a data accumulation and management system, using a stratified allocation method based on years of experience (five years or less and six years or more). Entries were closed when the number of participants reached 176. Once participants were automatically assigned to one of the groups, they and data managers were not blinded to the groups. Electronic questionnaires and intervention materials were delivered automatically at the preset time points. The analyst blindly analyzed the outcome measures.

### Study intervention

#### Program characteristics

This program was named YURAIKU-PRO. The program is a three-hour video in relation to an e-learning program for professionals to support parents with SPMI and their family members. The introduction (Module 0) and Modules 1–6 were divided into basic and specialized modules. The specific times and contents are listed in Table 1.

**Table 1:** Components of the program

Module	Minutes	Title	Contents	Aims	Main program goals					
General modules					1)	2)	3)	4)	5)	6)
0	17 min	Introduction	Explanation of the program, narration of a personal story by a parent with a mental disorder	Understand the program overview, learn about the parents' experience, be aware of professional stigma, and motivate them to learn.		X	X			
1	26 min	Mental illness and support system	Major mental illnesses, and the roles and collaboration of support organizations	Understand the basics of mental illness, be aware of professional stigma, understand the relationship between symptoms of mental illness and child care, and outline the role of support agencies.	X				X	X
2	20 min	Impact of mental disorders on parenting	Child-rearing difficulties caused by mental disorders, and how to support them	Understand child-rearing difficulties caused by mental disorders, and how to support them.	X			X		
3	28 min	Parents' experiences and support needs	Parents' experiences of difficulties and growth in raising their children; parents' experiences of receiving support and support needs	Understand parenting experiences and support needs of parents, and reduce professional stigma.		X	X	X		
4	37 min	Family-focused practice	Impacts on and support for children, spouses, and family relationships	Understand the importance of supporting children, their situations and feelings, and their support needs. Understand the difficulties and support needs that spouses are likely to have. Understand family relationships, support for the family as a whole, and support for each member.		X	X	X		
Specific modules for professionals in charge of maternal and child health or child welfare										
5	25 min	Initial stages of intervention	Crisis intervention, building therapeutic relationships, and roles of professionals in charge of maternal and child health or child welfare	Understand the role of professionals in charge of maternal and child health or child welfare. Understand specific methods of crisis intervention and therapeutic relationship building, and feel confident in their ability to provide support.			X	X	X	
6	32 min	Specific ways to provide support	Information on providing support during pregnancy, support to stabilize medical conditions and life, family-focused practice, and interagency collaboration	Understand how to support during pregnancy, support to stabilize medical conditions and life, support for the whole family, support in cooperation with related organizations, and feel confident in their ability to provide support.			X	X		X

Total 185 min

Program goals

1) childcare difficulties due to symptoms and characteristics of mental disorders

2) situation and feelings of the parents, spouses, and children

3) support needs of parents, spouses, and children

4) how to support the whole family

5) overview of the roles of maternal and child health, child welfare, and mental health welfare

6) how to collaborate with multiple institutions and professions

This program was developed through discussions in five meetings (for a total of eight hours), through e-mail among six parents with SPMI and seven multidisciplinary professionals who were research members of this project, including psychiatrists, public health nurses, psychiatric nurses, and mental health social workers. The six parents were core members of a self-help group called “YURAIKU” (meaning “relaxed parenting”). In addition, one spouse and two adult children of parents with SPMI who ran self-help groups participated in two meetings and provided comments. First, the author developed a draft video using the findings from previous interviews and a survey of parents with SPMI [47], their spouses [48], children [49], public health nurses [23], and child welfare workers [50]. Various opinions on most comments, including the structure and content of the video, were shared and reflected in the videos. For example, there was opposition from parents to the fact that professionals assessed it as not doing what they could not do, and from the spouse to the fact that some PHNs decided that home services were not needed, even if the spouse indicated a need for it. The comments reflected in the video are what was actually said. The children’s perspectives were addressed with minor modifications.

The e-learning program has five main characteristics. First, it focuses on the perspectives and support needs of parents with SPMI. Second, it is a family focused practice that supports an entire family. Third, it is a program with content for multiple professions in order to understand basic knowledge and skills. Fourth, it is based on experiences and support skills obtained through interviews and surveys of parents with SPMI and their children, spouses, and professionals to support them. Fifth, digital storytelling is used.

The goals were as follows:1) be able to describe the symptoms of mental illness and the difficulties in child rearing arising from the characteristics of mental disorders; 2) be able to describe the situation and feelings of the parents, spouses, and children involved; 3) be able to describe the support needs of the parents, spouses, and children involved; 4) be able to describe how to support the entire family; 5) be able to outline the roles of maternal and child health, child welfare, mental health, and medical welfare; and 6) be able to describe how to collaborate with multiple agencies and professionals. The program goals included knowledge and skills regarding parental SPMI (primarily goal 1), family-focused practice (primarily goals 2–4), parent and family situations, support needs for therapeutic relationship building (primarily goals 2–3), and interagency collaboration (primarily goals 5–6).

#### The program’s rational basis

The program was developed based on a training framework to deliver professional development programs in the area of parental SPMI [33]. The framework has a hierarchy and is divided into four tiers from general to targeted/specific training. Tier one is an anti-stigma message that aims to reduce the stigmatizing views of those with mental illness. Tier two is raising professionals’ awareness about the needs of parents and children in families where a parent has a mental illness. Tier three is a differentiated course for professionals, roles, and disciplines. Tier four is a training program for specific interventions. This program, in considering the anti-stigma messages in tier one, included the digital storytelling of a parent with SPMI (introduction) and verbatim descriptions of their experience regarding raising children (Module 3). As tier two aimed to raise the professionals’ awareness of the parents’ and children’s needs, understanding mental disorders (Module1), the impact of disorders on parenting (Module 2), family needs, and family-focused practices (Module 4) were included in this program. As tier three, Modules 5 and 6 are for professionals in charge of maternal and child health or welfare; and were developed in this program based on interviews that were conducted [23, 50]. Modules 5 and 6 include building therapeutic relationships and interagency collaboration. Digital stories in the animation style of junior PHNs consulting senior PHNs were used. Tier four was not included in the program because its purpose was to improve knowledge and skills regarding usual care, as opposed to specific intervention skills.

The learning process in each module was composed of Gagné’s instructional events [51]. To gain the necessary attention and provide feedback, questions about how to respond to cases were posed first, followed by how to respond, which was given after new knowledge was provided. Learning objectives, prerequisites, presentation of stimulus materials, and learning guidance were also provided. To retain and transfer what was learned, the text was sent as an e-mail attachment so that participants could use it as a resource. After viewing each module, the participants were asked to submit a brief report using an electronic questionnaire. They were asked to write about what they had learned to assess their own learning outcomes, reflect on their performance, and use their acquired knowledge for future performance.

### Analyses

The total study population was defined as the Intention-to-Treat (ITT) population. Two types of population analyses were performed. For the evaluation of outcomes in this trial, the full analysis set (FAS) was used, excluding the scores of those who did not respond to T1. The other type was the per-protocol analysis set (PPS), which excluded those in the FAS who did not respond on time, those who did not complete all modules before the deadline (six weeks) in the intervention group, and those who withdrew from the study.

First, descriptive statistics for FAS were calculated, and group comparisons between the intervention and control groups were made. Next, the total score on the Sense of Coping Difficulty/Possibility Scale at one-month post-intervention (endpoint, T3) as a primary outcome measure was calculated for the FAS, and the difference between groups in the mean was calculated by t-test and Cohen’s d was calculated as the effect size. As a sub-analysis, an analysis of covariance (ANCOVA) was performed using the baseline score (T1) and years of experience as covariates. The same analyses were performed for PPS. The same analysis as that for the total scores was performed for the two subscales of the Sense of Coping Difficulty/Possibility Scale.

To examine effectiveness over time, we fitted a mixed model for repeated measures with group, time point (T1, T2, and T3), group, and time interactions as fixed effects, and study participants as a random effect and analyzed the total scores and subscales. A mixed model for repeated measures, similar to the Sense of Coping Difficulty/Possibility Scale, was performed with scores on skills related to mental health issues and the sum score of achievement of program goals.

Process evaluations were analyzed in an exploratory manner only in the intervention group. To examine the relationship between the number of views and outcome measures, we used a linear model with the change from baseline as the dependent variable and the mean number of views and years of experience as independent variables. Short reports submitted by participants after viewing each module were reviewed by the researchers to determine if they contained anything that needed to be improved regarding the videos.

The missing data were not supplemented. A *p*-value of 0.05 or less was considered significant. All analyses were performed using SAS9.4 (SAS Institute Inc., Cary, NC, USA).

### Ethics

This study was approved by the Ethics Committee for the Intervention Study of Osaka University Hospital (approval no. 21237; 19/10/2021). Written informed consent was obtained from all participants. This study was conducted in accordance with the principles of the Declaration of Helsinki. The researchers did not reveal the identity of the participants to the health centers. In addition, the data were separated from personal information and managed with an ID. This project was registered in the Clinical Trial Registry (UMIN000045765; 1/11/2021). No adverse events were reported.

## Results

### Study participants

**Fig. 1:**
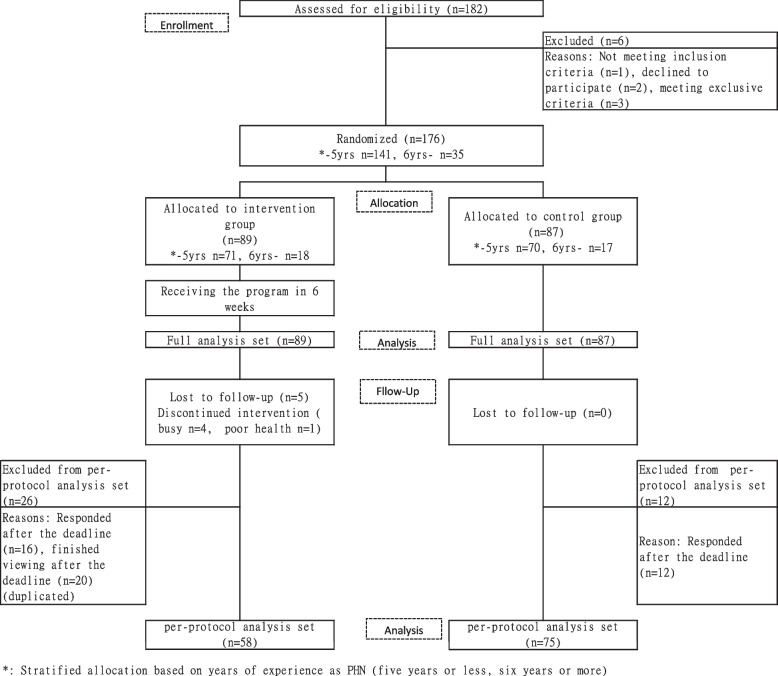
Flow of study participants

This study was conducted between November 2021 and March 2022. The flow of study participants is shown in Fig. 1. A total of 182 applied for the study; six were excluded because one did not meet the inclusion criteria, two declined to participate, and three could not conduct electronic questionnaires or video viewing operations due to technical issues (exclusion criteria). The recruitment ended when the planned sample size was reached. There were a total of 176 participants. 141 had five years or less of experience as a PHN, of which 71 were allocated to the intervention group and 70 to the control group. 35 participants had six or more years of experience as a PHN, out of which 18 were allocated to the intervention group and 17 to the control group. A total of 89 and 87 participants in the intervention and control groups, respectively, were analyzed for the FAS. In the intervention group, five participants withdrew from the study (four busy and one in poor health) after responding to T1. Moreover, 26 participants responded or finished viewing after the deadline, and 58 were categorized into the PPS group. In the control group, as nobody withdrew from the study and 12 responded after the deadline, 75 participants were categorized into the PPS group.

### Baseline characteristics of study participants

The demographics of the study participants in the intervention and control groups are shown in Table 2. The study participants’ years of experience as PHNs were short, averaging 3.7 and 3.4 years (intervention group and control group, respectively). Although most of them had supported parents with SPMI (91.0% and 87.4%, respectively), half of them had no learned experience about support for parents with SPMI. No statistically significant differences were observed between the intervention and the control groups.

**Table 2:** Baseline characteristics of study participants by group

Baseline characteristics		Intervention group (*n*=89)		Control group (*n*=87)		
		Mean/n	SD/%	Mean/n	SD/%	p
Age		28.6	5.1	28.1	5.4	0.240
Gender	Female	88	98.9%	87	100.0%	1.000
	Male	1	1.1%	0	0.0%	
Years of experience as a PHN		3.7	2.3	3.4	2.2	0.283
Experience in charge of mental health services	Yes	19	21.3%	16	18.4%	0.707
	No	70	78.7%	71	81.6%	
Years of experience in charge of maternal and child health		3.0	2.0	3.1	2.2	0.899
Experience of supporting parents with SPMI	Yes	81	91.0%	76	87.4%	0.475
	No	8	9.0%	11	12.6%	
Experience of learning about support for parents with SPMI	Yes	44	49.4%	35	40.2%	0.230
	No	45	50.6%	52	59.8%	
Experience of supporting people with SPMI who are not raising children	Yes	51	57.3%	56	64.4%	0.358
	No	38	42.7%	31	35.6%	

^*^P-values:Wilcoxon rank sum test for continuous variables, Fisher's exact test for categorical variables

SPMI: Severe and persistent mental illness

### Outcome measures at endpoint (T3)

Analysis results for the total score and two subscales of the Sense of Coping Difficulty/Possibility Scale at endpoint (T3) in Intent to Treat Analysis (FAS) are shown in Table 3. In the intervention group, the total score at endpoint (T3) was significantly higher than that in the control group by t-test for both FAS and PPS, as well as ANOVA with covariates of baseline score and years of experience as a PHN on both FAS and PPS. In addition to the total score, the scores of the subscales at T3 were also significantly higher than those of the control groups by both the t-test and ANOVA. The effect size (Cohen’s d) of the total score was 1.19 (FAS) and 1.12 (PPS), and that of the Sense of Coping Difficulty subscale was 1.27 (FAS), indicating large enough. The effect size of the Sense of Coping Possibility subscale was 0.65 (FAS), indicating a medium.

**Table 3:** Main outcomes at endpoint(T3) (Intent to Treat Analysis)

		Endpoint (T3)		Effect size	Difference (I-C)			
		Mean	SD	Cohen's d	Mean Difference	95% CI	*p*-value t-test	*p*-value ANOVA*
Sense of Coping Difficulty/Possibility Scale	Intervention (*n*=84)	29.2	2.9	1.19	3.32	2.29, 4.34	<.001	<.001
	Control (*n*=87)	25.8	2.7					
Sense of Coping Difficulty subscale	Intervention (*n*=84)	13.4	1.9	1.27	2.38	1.82, 2.95	<.001	<.001
	Control (*n*=87)	11.1	1.9					
Sense of Coping Possibility subscale	Intervention (*n*=84)	15.7	1.6	0.65	0.96	0.51, 1.40	<.001	<.001
	Control (*n*=87)	14.8	1.3					

I-C: Intervention and control groups

^*^ covariates: baseline score and years of experiences as a PHN

### Effectiveness over time

**Fig. 2:**
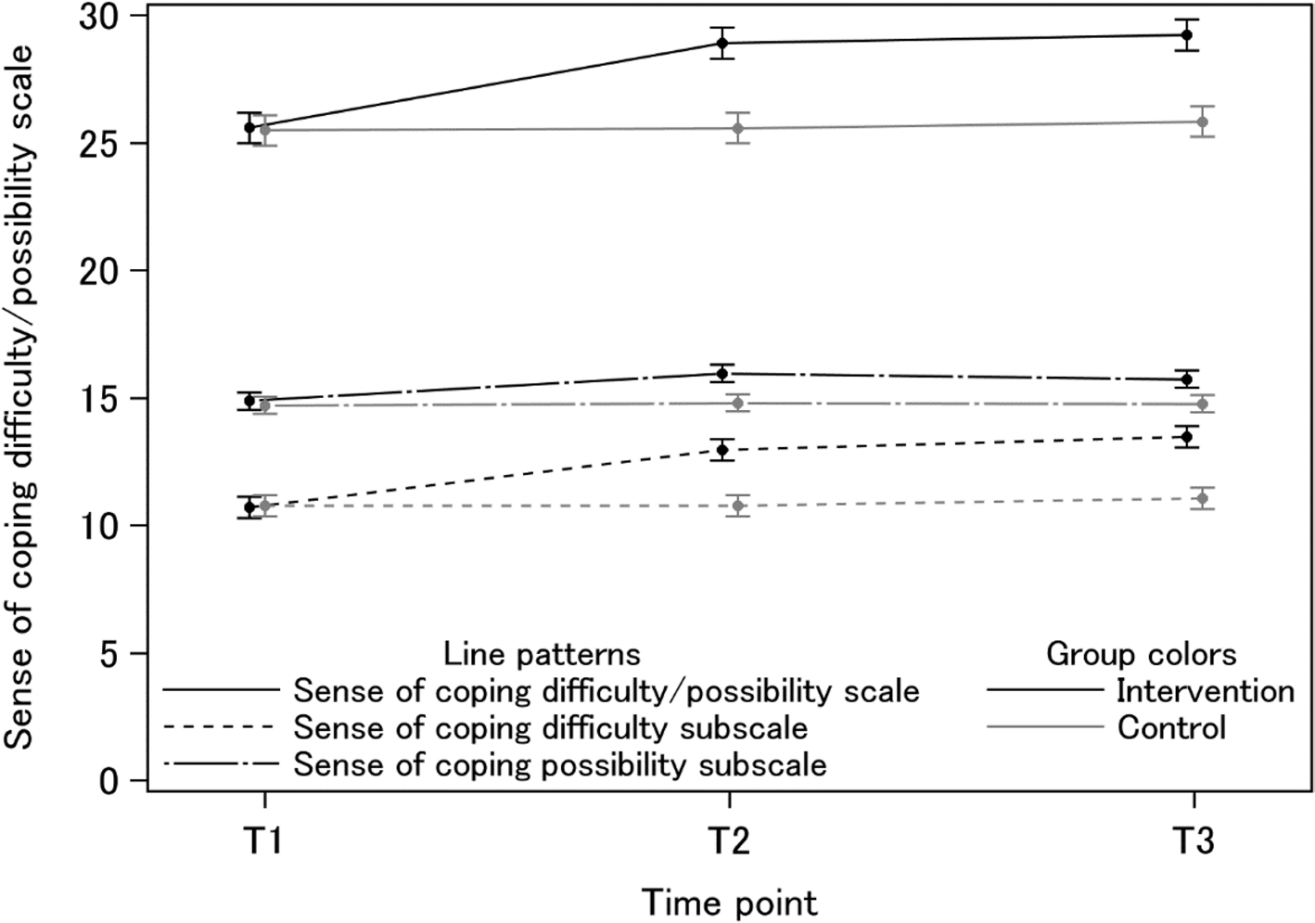
Interaction of time and intervention as T1, T2 and T3

As shown in Table 4, the analysis results of a mixed model for repeated measures showed that group and time interactions on total scores of the Sense of Coping Difficulty/Possibility Scale and the subscales (shown in Figure 2), skills related to mental health issues, and the sum score of program goal achievement were not significantly different at T1 but were significantly different at T2 and T3.

**Table 4:** Interaction of time and intervention as T1, T2 and T3 (Intent to Treat Analysis)

		Baseline (T1)		Post (T2)		Endpoint (T3)		group×T2	group×T3
		Mean	SD	Mean	SD	Mean	SD	p	p
Sense of Coping Difficulty/Possibility Scale	Intervention (*n*=89)	25.6	2.9	28.9	3.2	29.2	2.9	<.001	<.001
	Control (*n*=87)	25.5	2.6	25.6	2.7	25.8	2.7		
Sense of Coping Difficulty subscale	Intervention (*n*=89)	10.7	2.1	12.9	2.3	13.4	1.9	<.001	<.001
	Control (*n*=87)	10.8	1.7	10.8	1.9	11.1	1.9		
Sense of Coping Possibility subscale	Intervention (*n*=89)	14.9	1.8	16.0	1.8	15.7	1.6	<.001	0.001
	Control (*n*=87)	14.7	1.5	14.8	1.4	14.8	1.3		
Skills related to mental health issues	Intervention (*n*=89)	1.45	1.18	2.94	1.23	3.24	1.15	<.001	<.001
	Control (*n*=87)	1.52	1.23	1.72	1.34	2.03	1.21		
Achievement on program goals		Median	IQR	Median	IQR	Median	IQR		
1) childcare difficulties due to symptoms and characteristics of mental disorders	Intervention (*n*=89)	3	3-4	5	4-5	5	4-5	n/a	n/a
	Control (*n*=87)	3	2-4	3	3-4	4	3-4		
2) situation and feelings of the parents, spouses, and children	Intervention (*n*=89)	3	2-4	5	4-5	5	4-5	n/a	n/a
	Control (*n*=87)	3	2-4	3	2-4	4	3-4		
3) support needs of parents, spouses, and children	Intervention (*n*=89)	3	2-4	5	4-5	5	4-5	n/a	n/a
	Control (*n*=87)	3	2-4	3	3-4	3	3-4		
4) how to support the whole family	Intervention (*n*=89)	3	2-4	4	4-5	4	4-5	n/a	n/a
	Control (*n*=87)	3	2-3	3	2-4	3	3-4		
5) overview of the roles of maternal and child health, child welfare, and mental health welfare	Intervention (*n*=89)	3	2-4	4	4-5	5	4-5	n/a	n/a
	Control (*n*=87)	3	2-4	3	2-4	3	3-4		
6) how to collaborate with multiple institutions and professions	Intervention (*n*=89)	3	2-4	5	4-5	5	4-5	n/a	n/a
	Control (*n*=87)	3	2-4	3	3-4	4	3-4		
Sum of 1) to 6)	Intervention (*n*=89)	18	14-22	27	23-30	27	24-30	<.001	<.001
	Control (*n*=87)	18	13-23	20	16-24	21	16-26		

p: repeated measures mixed-effects model with group, time point (T1, T2, and T3), group and time interactions as fixed effects

IQR: interquartile range; n/a: not applicable

### Process and feasibility evaluations

The number of views for each module in the intervention group is presented in Table 5. The most common viewing frequency for all modules was twice (55.1–61.8%), followed by once (23.6–30.3%). There was no significant relationship between the number of views and outcome measures.

**Table 5:** Number of views each module

Module	Title	0 time	1 time	2 times	3 times	4 times	5 times
General modules		n (%)	n (%)	n (%)	n (%)	n (%)	n (%)
0	Introduction	1 (1.1%)	27 (30.3%)	50 (56.2%)	10 (11.2%)	1 (1.1%)	0 (0.0%)
1	Mental illness and support system	1 (1.1%)	26 (29.2%)	49 (55.1%)	10 (11.2%)	3 (3.4%)	0 (0.0%)
2	Impact of mental disorders on parenting	1 (1.1%)	23 (25.8%)	54 (60.7%)	9 (10.1%)	2 (2.2%)	0 (0.0%)
3	Parents' experiences and support needs	3 (3.4%)	24 (27%)	55 (61.8%)	7 (7.9%)	0 (0.0%)	0 (0.0%)
4	Family-focused practice	4 (4.5%)	23 (25.8%)	52 (58.4%)	8 (9%)	1 (1.1%)	1 (1.1%)
Specific modules for professionals in charge of maternal and child health or child welfare							
5	Initial stages of intervention	5 (5.6%)	21 (23.6%)	51 (57.3%)	10 (11.2%)	2 (2.2%)	0 (0.0%)
6	Specific ways to provide support	5 (5.6%)	21 (23.6%)	52 (58.4%)	9 (10.1%)	2 (2.2%)	0 (0.0%)

*n*=89

Of the 84 participants in the intervention group who finished all modules, 17 (20.2%) answered “long,” 67 (79.8%) answered “just right,” and none answered “short” for the length of the program.

## Discussion

### Effectiveness of the program

In the intervention group, the total score and scores of the two subscales of the Sense of Coping Difficulty/Possibility Scale at the endpoint (T3) were significantly higher than those of the control group. Moreover, the analysis results of a mixed model for repeated measures showed that the group and time interactions on total scores of the Sense of Coping Difficulty/Possibility Scale and the subscales, skills related to mental health issues, and the sum score of the program goal achievements were not significantly different at T1, but were significantly different at T2 and T3. This result indicates that significant effects were observed over time up to one month after the intervention. The program achieved its goals and was as effective as intended.

The effect size was moderate (Cohen’s d=0.65) for the Sense of Coping Possibility subscale, whereas it was large enough (Cohen’s d=1.27) for the Sense of Coping Difficulty subscale [43]. In a survey of municipal PHNs in Japan, 91.1% of PHNs responded that support for people with SPMI was important, whereas only 10.8% were not confused about supporting them, and only 26.9% supported them as not difficult [52]. Therefore, providing support to parents with SPMI is considered a challenge for PHNs in Japan. In this program, many support methods have been incorporated, based on practical examples. This may have led to a better understanding of the parents and support methods, and reduced the difficulty in providing support. This e-learning program was particularly effective in reducing PHNs’ difficulties in supporting parents with SPMI.

The Sense of Coping Possibility subscale also had a moderate effect size, but the effect persisted until one month later. This measure may also reflect a change in PHNs toward positive attitudes. We believe that digital storytelling and incorporation of verbatim descriptions by parents with SPMI are effective.

Skills related to mental health issues also showed a sustained effect up to one month after the intervention. This indicator is a self-assessment measure of knowledge, support skills, and support experience. It can be concluded overall that the participants gained knowledge and skills.

The sum score of program goal achievement also showed a sustained effect up to one month after the intervention. Program goal achievement was based on the ability to explain their knowledge and skills in parental SPMI, family-focused practice, parent and family situations, support needs necessary for therapeutic relationship-building, and interagency collaboration. Overall goals were achieved through this program.

### Learning style and feasibility of the program

In this study, an e-learning program was evaluated using an RCT. Many e-learning programs for continuing education for nurses are primarily used to improve nurses’ knowledge, skills, and self-efficacy [36], and we believe that this was a suitable method that met the objectives of this study. In this program, a parent with SPMI narrated her story. Furthermore, an animation in the form of junior PHNs consulting with senior PHNs was used. According to a systematic review article on digital storytelling in health profession education, the effectiveness of using only patients is limited, and the use of health professionals’ stories enhances learning [39]. Although the styles of the digital stories in this program were different between patients and PHNs, both styles may be effective in using digital storytelling in nursing education.

It has been reported that e-learning programs for professional development exist for parents and families; however, few have been evaluated for their effectiveness [33]. Two e-learning training programs “Keeping Families and Children in Mind” [41] and “Let’s Talk about Children” [42] include knowledge about parents with SPMI, family-focused practice, and other contents similar to YURAIKU-PRO. Mental health professionals primarily use these programs. They include case studies that were not included in the YURAIKU-PRO and are, therefore, more advanced than the YURAIKU-PRO. The YURAIKU-PRO is suitable for nurses in maternal and child health areas, who do not have specialized mental health knowledge or skills.

The program also worked well as an e-learning program one month after its completion. One reason for the continued effectiveness of the program may be its learning style, which was divided into six modules. Although the modules were short, it is possible that the effect was sustained by repeated self-reflection and action planning each time using Gagné’s instruction [51]. Of the 89 participants in the intervention group, all but five completed the six modules during the COVID-19 pandemic despite the lack of incentives, such as cooperation rewards. This indicates a high level of motivation for learning. Approximately 70% of participants viewed each module at least twice. To date, no e-learning program has focused on this subject in Japan, although face-to-face training programs have focused on the perinatal period [53]. Even though the e-learning program was only about three hours long, the content was sufficiently effective for PHNs with less than ten years of experience. About 80% responded that the program was just right, and 20% responded that it was too long; suggesting that a shorter program may be more suitable for busy field workers.

### Study limitations

This study has several limitations. First, the effectiveness evaluation was not available until after one month, making evaluation over a longer time period impossible. Next, as we measured self-efficacy for support, we did not know whether the study participants could successfully support them. Third, significant effects of the program were found, even after adjusting for years of experience as a PHN. This program may be effective for PHNs with more than ten years of experience, which was not the subject of this study. Fourth, owing to the data management system used in the study, it was not possible to determine if the video was actually viewed all the way through or for how long.

Finally, a potential bias is that many participants may have worked in workplaces that understood the training, or were studious. Thus, a shorter program may be appropriate for those who are less interested in studying or who cannot view it during their working hours, but only during their private time. Possible research directions include shorter programs, long-term evaluation of effectiveness, inclusion of PHNs with more than ten years of experience, and data analysis, including bounce rates and viewing time for each module.

The recommended method for viewing this program is not to view all modules at once but to reflect and practice daily each time one module is viewed. This repetition is believed to result in higher retention of knowledge and skills.

A possible direction for future research is to develop effective training programs for professionals other than PHNs. Training programs for childcare workers and mental health professionals appropriate for their respective specialties should be developed.

## Conclusion

The e-learning program YURAIKU-PRO was found to be effective, particularly in reducing PHNs' difficulties in supporting parents with SPMI. In addition to the knowledge and skills related to parental SPMI, family-focused practices, therapeutic relationship building, and interagency collaboration are especially important when supporting parents with SPMI. The use of digital storytelling through e-learning is effective and recommended as a training style for nurses.

## Data Availability

Access to the materials for this program will soon be made openly available. These data are available from the corresponding author upon request. The datasets generated and analyzed during the current study are not publicly available because of confidentiality concerns but are available from the corresponding author upon reasonable request after the approval of the institutional ethics review committee.

## Abbreviations

ANOVA: Analysis of variance
FAS: Full analysis set
ITT: Intention-to-treat
PCA: Principal component analysis
PHN: Public health nurse
PPS: Per-protocol analysis set
RCT: Randomized controlled trial
SPMI: Severe and persistent mental illness
